# Treatment of Dengue

**Published:** 1890-03-29

**Authors:** 


### TREATMENT OF DENGUE.

We gave recently an account of the progress of the epidemic of last year in the Levant, with a description of the symptoms, &c., of the disease, as well as of the treatment adopted by Dr. De Brun. We may now add a few more suggestions contributed by the Greek physicians—Drs. Karageorgiades, Chrysochoos, and Siotis. They agree with De Brun as to the uselessness of quinine, which too, adds cinchonism to the aural disturbance already painfully present, and the value of antipyrin in relieving the headache and pains, as it does in influenza; but they prefer small doses of 4—8 centigrams only every hour or two, combined with Tr. Belladonna m. v-x. They give chloral to induce sleep, acid fruit drinks, as tamarind water, &c., for the thirst, milk with lime or soda water, &c. Dr. Karageorgiades, too, finds that an infusion of jaborandi leaves, one pint to twenty of water, given in half-ounce doses every two hours, is very efficient in reducing the fever.

